# Combined telemonitoring and telecoaching for heart failure improves outcome

**DOI:** 10.1038/s41746-023-00942-4

**Published:** 2023-10-17

**Authors:** Katharina Knoll, Stefanie Rosner, Stefan Gross, Dino Dittrich, Carsten Lennerz, Teresa Trenkwalder, Stefanie Schmitz, Stefan Sauer, Christian Hentschke, Marcus Dörr, Christian Kloss, Heribert Schunkert, Wibke Reinhard

**Affiliations:** 1 German Heart Centre Munich, Department of Cardiology, Technical University Munich, Munich, Germany; 2 DZHK (German Centre for Cardiovascular Research), partner site Munich Heart Alliance, Munich, Germany; 3 Department of Internal Medicine B, University Medicine Greifswald, Greifswald, Germany; 4 DZHK (German Centre for Cardiovascular Research), partner site Greifswald, Greifswald, Germany; 5 Health Care Systems GmbH (HCSG), Pullach im Isartal, Germany; 6 Krankenkasse KNAPPSCHAFT, Bochum, Germany; 7 Novartis Pharma GmbH, Nürnberg, Germany

**Keywords:** Outcomes research, Patient education, Cardiomyopathies

## Abstract

Telemedicine has been shown to improve the outcome of heart failure (HF) patients in addition to medical and device therapy. We investigate the effectiveness of a comprehensive telehealth programme in patients with recent hospitalisation for HF on subsequent HF hospitalisations and mortality compared to usual care in a real-world setting. The telehealth programme consists of daily remote telemonitoring of HF signs/symptoms and regular individualised telecoaching sessions. Between January 2018 and September 2020, 119,715 patients of a German health insurer were hospitalised for HF and were eligible for participation in the programme. Finally, 6065 HF patients at high risk for re-hospitalisation were enroled. Participants were retrospectively compared to a propensity score matched usual care group (*n* = 6065). Median follow-up was 442 days (IQR 309–681). Data from the health insurer was used to evaluate outcomes. After one year, the number of hospitalisations for HF (17.9 vs. 21.8 per 100 patient years, *p* < 0.001), all-cause hospitalisations (129.0 vs. 133.2 per 100 patient years, *p* = 0.015), and the respective days spent in hospital (2.0 vs. 2.6 days per year, *p* < 0.001, and 12.0 vs. 13.4, *p* < 0.001, respectively) were significantly lower in the telehealth than in the usual care group. Moreover, participation in the telehealth programme was related to a significant reduction in all-cause mortality compared to usual care (5.8 vs. 11.0 %, *p* < 0.001). In a real-life setting of ambulatory HF patients at high risk for re-hospitalisation, participation in a comprehensive telehealth programme was related to a reduction of HF hospitalisations and all-cause mortality compared to usual care.

## Introduction

The global burden of chronic heart failure (HF) is rising. Besides reducing the patient’s quality of life, HF is associated with frequent hospitalisations and high morbidity and mortality rates^1^. Apart from poor prognosis, HF poses a substantial economic burden on health care systems^1^. Despite recent advances in drug therapy^1^, management of HF patients remains challenging and multimodal. On top of optimal medical and device therapy, telemedical interventions, especially non-invasive telemonitoring, are recommended by current ESC guidelines to reduce risk of recurrent HF and cardiovascular hospitalisations as well as cardiovascular deaths^1^.

Telehealth summarises a broad spectrum of interventions and encompasses synchronous patient-clinician communication (telephone or video visit), remote patient monitoring (telemonitoring) and self-management support (telecoaching)^2^. Besides providing continuity of care through virtual consultations with health care professionals (physicians or telenurses), telehealth can motivate patients to improve adherence to prognostically relevant medications and health promoting behaviour^3^. Remote monitoring can help to detect early signs of clinical deterioration avoiding delays in treatment adaptation, possibly avoiding hospitalisation^4^.

Several meta-analyses^5–10^ and observational studies^11,12^ as well as randomised controlled trials^13–15^ suggest clinical benefits from telehealth interventions on HF hospitalisations and/or mortality, respectively. However, some prospective clinical trials fail to prove advantages from their telehealth interventions^16–21^. The conflicting evidence is likely due to the heterogeneity of the telehealth interventions analysed and differences in patient populations^5^. Some authors assume additional benefit from more complex telemonitoring and telecoaching programmes^9^. The value of telehealth in real-life is even less understood. The aim of our study was to investigate the effectiveness of a telehealth programme combining telemonitoring and telecoaching for patients with a recent hospitalisation for HF on subsequent hospitalisations and mortality compared to usual care in a real-life setting through a retrospective, propensity score matched analysis.

## Results

### Study population

In this study, 6065 HF patients at high risk for re-hospitalisation and participating in a telehealth programme (telehealth intervention group, TH) were retrospectively compared to an equally sized propensity score matched usual care group (usual care control group, UC).

Table 1 compares the baseline characteristics of both groups at the time of screening. There were only small differences in the baseline characteristics of the TH and the propensity score matched UC group (Table 1 and Supplementary Table 3). Sensitivity analysis of other matching algorithms showed similar results (Supplementary methods, Supplementary Tables 1 and 2).

**Table 1:** Baseline characteristics from insurance claims data.

Metric	Telehealth intervention group	Usual-care control group	Cohen’s d
Number of patients	6065	6065	–
Dimensions exactly matched			
Gender (male)	3800 (62.7%)	3800 (62.7%)	–
Age groups			
Younger than 70 years	1469 (24.2%)	1469 (24.2%)	–
70–76 years	1458 (24.0%)	1458 (24.0%)	–
77–81 years	1710 (28.2%)	1710 (28.2%)	–
82 years and older	1428 (23.5%)	1428 (23.5%)	–
Main diagnosis at hospitalisation immediately preceding screening			
Heart failure^a^	2831 (46.7%)	2831 (46.7%)	–
Cardiovascular excluding heart failure^b^	2141 (35.3%)	2141 (35.3%)	–
All other	1093 (18.0%)	1093 (18.0%)	–
Proximity scores			
Proximity score base model	51.3% ± 28.5%	51.2% ± 28.4%	
Baseline characteristics not used for matching			
Age in years	75.3 ± 8.9	75.4 ± 8.9	0.016
Prospective one-year LoH (ACRA-LoH)	54.3% ± 14.5%	55.5% ± 13.4%	0.085
Hospitalisations, during the last 12 months prior to screening			
Number of all-cause hospitalisations	2.14 ± 1.64	2.08 ± 1.66	0.035
Number with main diagnosis heart failure^a^	0.46 ± 0.67	0.43 ± 0.66	0.058
Number of all hospital diagnoses (main and secondary diagnoses)	23.8 ± 22.0	23.4 ± 21.9	0.017
Time since last preceding hospitalisation	161 ± 110	159 ± 110	0.018
Days in hospital during previous 12 months	18.8 ± 20.6	18.5 ± 20.0	0.015
Medication during the last 12 months prior to screening			
Number of prescriptions	87.2 ± 48.1	87.5 ± 48.1	0.008
Proportion with ACE-inhibiters or ARBs	84.3%	82.1%	0.058
Proportion with ARNI	7.5%	7.4%	0.003
Proportion with beta-blockers	83.0%	81.7%	0.033
Proportion with diuretics	84.2%	83.8%	0.011
Proportion with MRA	33.9%	33.4%	0.012
Comorbidities during the last 12 months prior to screening			
Hypertension	82.6%	81.5%	0.028
Coronary heart disease	55.9%	55.1%	0.018
Stroke	2.1%	2.3%	0.011
Kidney disease	39.8%	41.2%	0.028
Diabetes mellitus	30.4%	32.0%	0.034
COPD and/or Asthma	48.6%	48.7%	0.002
Malignant diseases	8.1%	9.1%	0.035

Values are presented as absolute numbers ± standard deviation of as proportions (in %).

^a^Heart failure defined as ICD-10 Codes I50.*, I11.0*, I13.0*, I42.0*.

^b^Cardiovascular excluding heart failure defined as ICD-10 Codes I* excluding ICD-10 Codes associated to heart failure. Cohen’s d is a measure of effect size where values < 0.2 indicate small effects^31^.

**Fig. 1:**
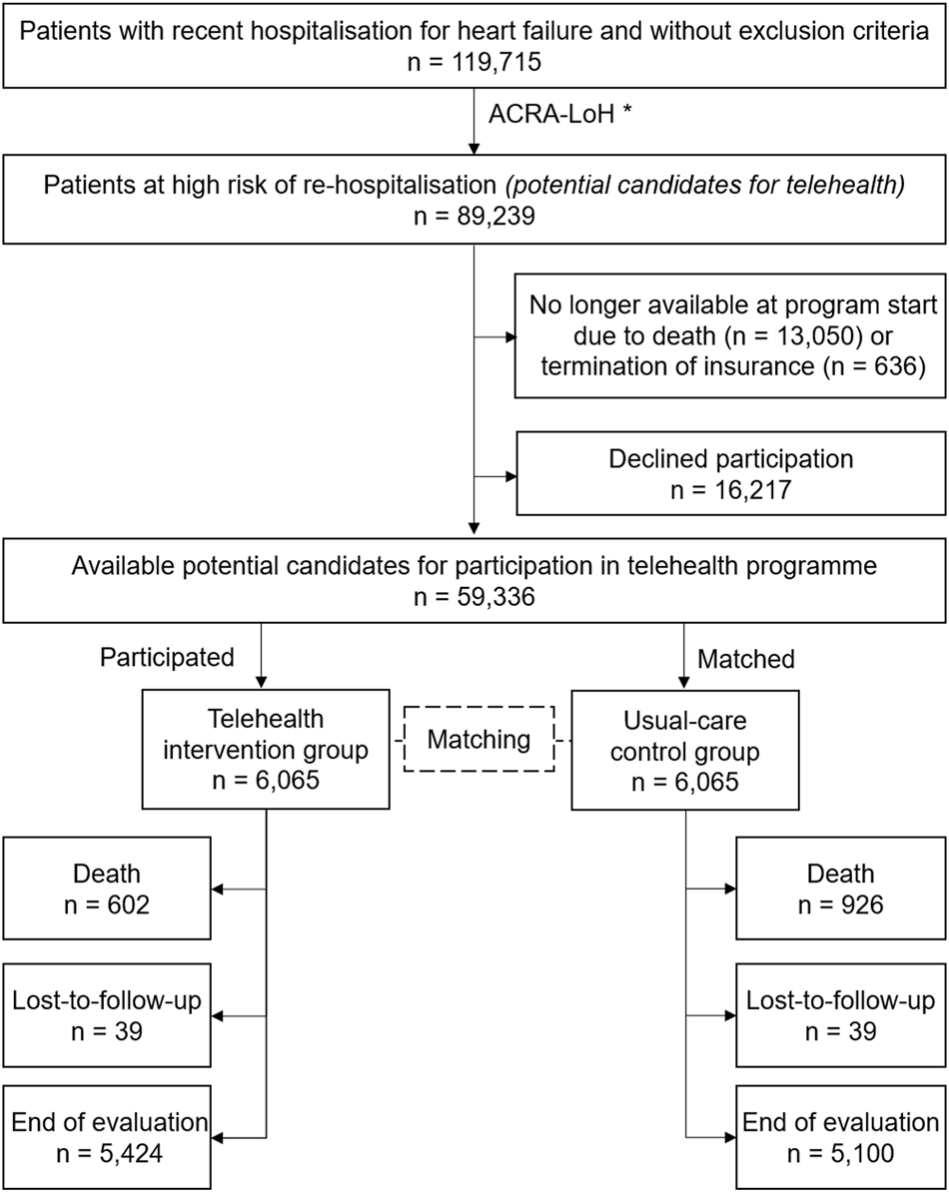
Study flow. *ACRA-LoH = likelihood of hospitalisation (LoH) calculated with the adaptable, comprehensive, risk assessment methodology (ACRA) based on historical data of patients from the insurance company.

In the intention-to-treat analysis (ITT), 602 patients of the TH group died and 39 were lost to follow-up (i.e., left the health insurance company). In the UC group 926 patients died during the evaluation period and 39 were lost to follow-up. The average evaluation period was 500 days (median 442 days, interquartile range (IQR) 309–681, Fig. 1).

In the on-treatment analysis (OT) the average on-treatment evaluation length was 441 days (median 409 days, IQR 255–606). During this period 402 patients died in the TH group, 24 were lost to follow-up and 1101 patients dropped out of the telehealth programme. Of these dropouts, 200 died after quitting the programme, 15 were lost to follow-up. For the remaining 886 TH patients, data was available until the end of the evaluation period (a detailed description of study dropouts is provided in the supplementary results). Out of 6065 patients in the UC group, 804 patients died and 34 were lost to follow-up.

### Primary outcome

**Fig. 2:**
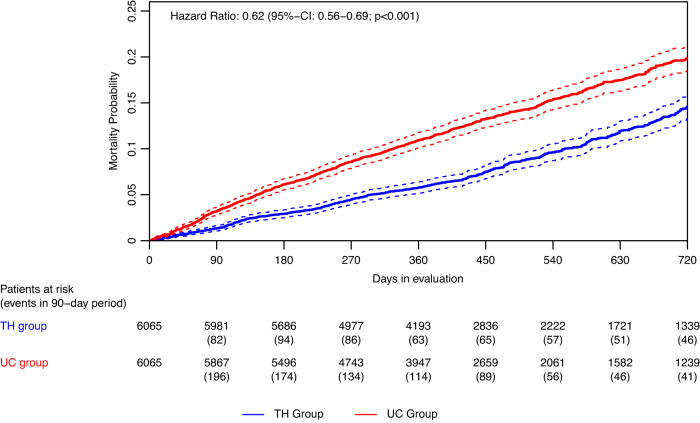
Kaplan–Meier-plots of the all-cause mortality probability (continuous line) with 95%-confidence interval (dotted line) in the intention-to-treat-analysis. Hazard ratio 0.62, 95%-confidence interval: 0.56–0.69, *p* < 0.001, Wald-test. Blue line: Telehealth group (TH). Red line: Usual care group (UC).

In the ITT analysis, all-cause mortality probability after one year was 5.8% (95%-confidence interval (CI): 5.2–6.4%) in the TH group compared to 11.0% (CI: 10.2–11.8%) in the propensity score matched UC group (*p* < 0.001, z-test, Fig. 2). Similarly, two year-mortality probability was 14.7% (CI: 13.4–16.0%) in the TH group vs. 20.2% (CI: 18.8–21.6%) in the UC group (*p* < 0.001, z-test).

In the OT analysis, all-cause mortality probability after one year was 4.8% (CI: 4.2–5.4%) in the TH group vs. 11.0% (CI: 10.1–11.9%) in the UC group (*p* < 0.001, z-test, Supplementary fig. 1), and the two year-mortality probability was 10.9% (CI: 9.7–12.2%) vs. 19.8% (CI: 18.3–21.4%), respectively (*p* < 0.001, z-test).

The Hazard ratio (HR) for all-cause mortality probability was 0.62 (CI, 0.56–0.69; *p* < 0.001, Wald-test, Fig. 2) in the ITT and 0.47 (CI, 0.42–0.53; *p* < 0.001, Wald-test, Supplementary Fig. 1) in the OT analysis. The number needed to treat to prevent one death after one year was 19.3 in the ITT and 16.1 in the OT analysis, respectively.

### Secondary outcomes

The number of hospital days per year were significantly lower in the TH group compared to the UC group for all main diagnoses (HF, cardiovascular and all-cause) in both ITT and OT analysis. Moreover, the number of HF hospitalisations and all-cause hospitalisation were also significantly lower in the TH compared to the UC group in both analyses (Table 2).

**Table 2:** Secondary outcomes: Number and duration of hospitalisations for specific main diagnoses of the telehealth intervention (TH) and usual care control (UC) group.

	Intention-to-treat-analysis			On-treatment-analysis		
	Telehealth group	Usual-care group	*p*-value	Telehealth group	Usual-care group	*p*-value
Number of patients	6065	6065	–	6065	6065	–
Days alive out of hospital (%)	90.7%	86.3%	<0.001			
Hospitalisations (per 100 patient years)						
- All-cause	129.6 (125.3–133.9)	133.2 (128.8–137.8)	0.015	125.7 (121.4–130.1)	133.6 (129.0–138.3)	0.012
- Cardiovascular	47.4 (45.1–49.7)	48.3 (46.0–50.6)	0.164	46.2 (43.9–48.6)	47.8 (45.4–50.3)	0.213
- Heart failure	17.9 (16.5–19.3)	21.8 (20.2–23.3)	<0.001	16.7 (15.3–18.0)	21.3 (19.8–22.9)	<0.001
Days in hospital (per year)						
- All-cause	12.0 (11.5–12.5)	13.4 (12.8–13.9)	<0.001	11.4 (10.9–11.9)	13.3 (12.7–13.9)	<0.001
- Cardiovascular	4.3 (4.1–4.6)	4.9 (4.6–5.2)	<0.001	4.1 (3.9–4.4)	4.8 (4.5–5.1)	<0.001
- Heart failure	2.0 (1.8–2.1)	2.6 (2.4–2.8)	<0.001	1.8 (1.6–2.0)	2.5 (2.3–2.7)	<0.001

All *p*-values below 0.05 remained below 0.05 after adjustment for multiple comparisons. *P*-values from *t*-test (days alive and out of hospital) and Wald-test (hospitalisations per 100 patient years and days in hospital per year).

Over the evaluation period, the percentage of days hospitalised and days alive outside hospital (DAOH) of the TH group was 90.7% (CI: 90.1–91.3%); this was significantly higher than in the UC group (86.3%; CI: 85.5–87.0%, *p* < 0.001, *t*-test).

**Fig. 3:**
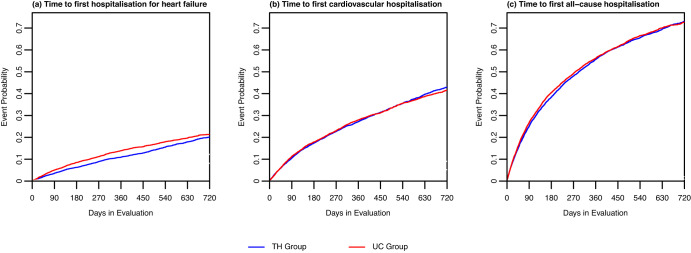
Competing risk curve for time to first hospitalisation with specific main diagnosis and death as competing risk in the telehealth intervention (TH) and usual care (UC) group. **a** Hospitalisation with main diagnosis heart failure, **b** Hospitalisation with main diagnosis cardiovascular disease, **c** All-cause hospitalisations. Blue line: Telehealth group. Red line: Usual care group.

The results of the competing risk analysis are depicted in Fig. 3. Competing risk curves of the time-to-event analysis show that time until first hospitalisation for HF was significantly longer in the TH than the UC group (*p* < 0.001,Wald-test, Fig. 3a). In contrast, there was no difference in the time until first cardiovascular or all-cause hospitalisation between the TH and UC group (*p* = 0.499 and *p* = 0.107 of Wald-test, respectively, Fig. 3b, c).

**Fig. 4:**
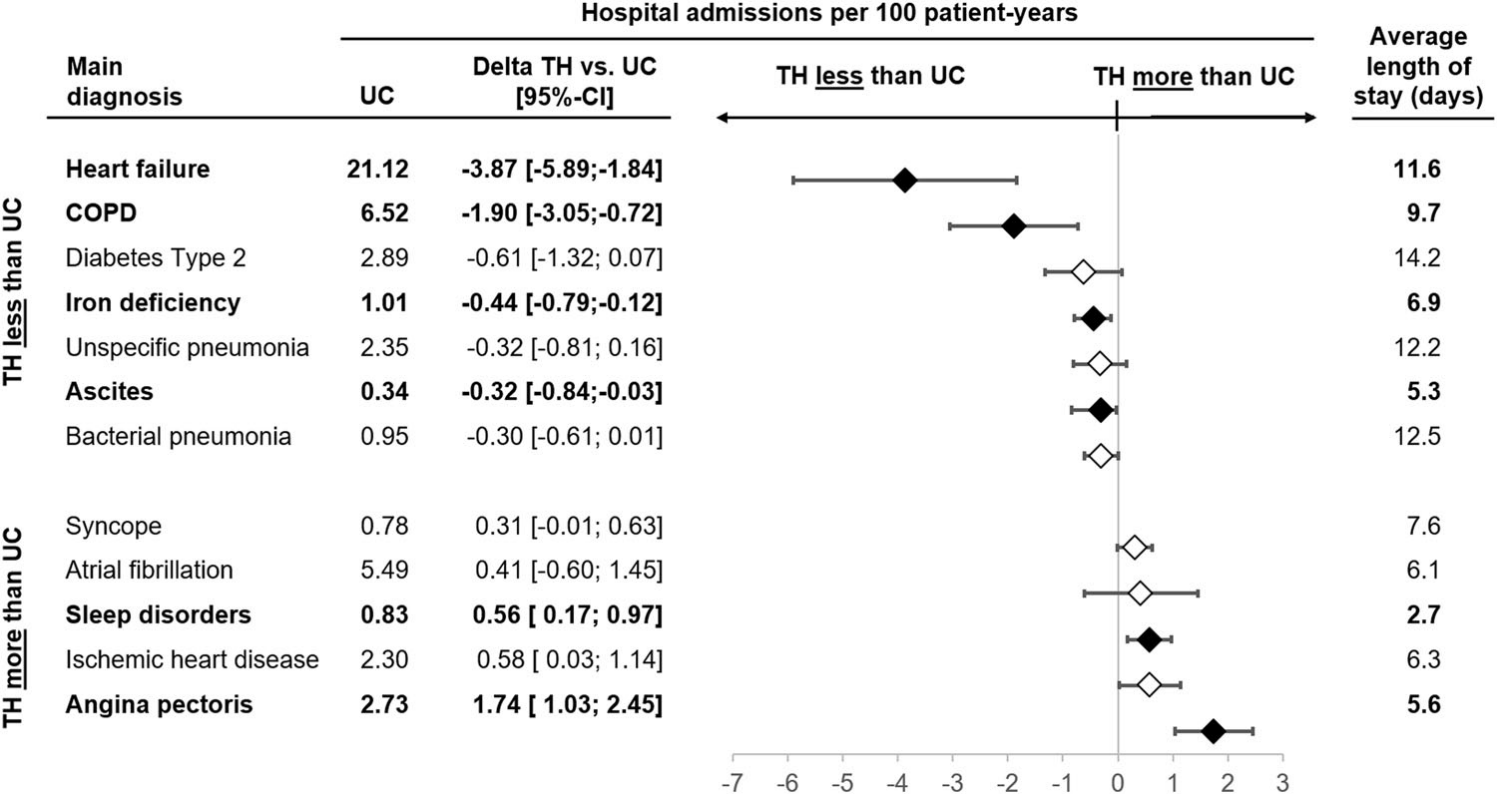
Differences in hospitalisations rates (per 100 patient years with 95%-confidence interval) and average length of stay for different main diagnoses of hospitalisation in telehealth (TH) versus usual care (UC) group. Solid diamonds and bold diagnoses indicate statistical significance (*p* < 0.05 after adjustment for multiple comparisons, Wald-test), empty diamonds statistically non-significant diagnoses (*p* > 0.05 after adjustment for multiple comparisons, Wald-test). The bars indicate the 95%-confidence interval for the differences.

The effect on hospitalisation was primarily driven by a reduction of hospitalisations due to HF, whereas hospitalisations for angina pectoris, stable chronic ischaemic heart disease and supraventricular arrhythmias were numerically higher in the TH group than in the UC group (Fig. 4). Admissions for other relevant comorbidities as main diagnosis were also reduced in the TH compared to the UC group (Fig. 4).

### Patient characteristics during the telehealth interventions

Over the duration of the study, the risk profile of the UC and TH group changed as the mortality in the UC group was higher and disproportionally affected high-risk patients, e.g. older patients and those with higher likelihood of hospitalisation (ACRA-LoH). Mean age increased by 0.53 years in the UC versus 0.79 years in the TH group after one year and 1.39 versus 1.99 years after two years, respectively. Similarly, the initial ACRA-LoH decreased by 1.0% after one year and by 2.6% after two years in the UC group, while in the TH group the initial ACRA-LoH decreased by 0.2% and 0.1%, respectively.

### Adherence and self-care

Adherence to the telemonitoring aspect of the telehealth programme was high with 81.9%. The adherence was high across all age groups (≤70 years: 78.1%, 70–76; 82.9%, 77–81: 84.5%, ≥82: 82.0%) and was slightly higher in men than in women (82.7% vs. 80.5%).

**Fig. 5:**
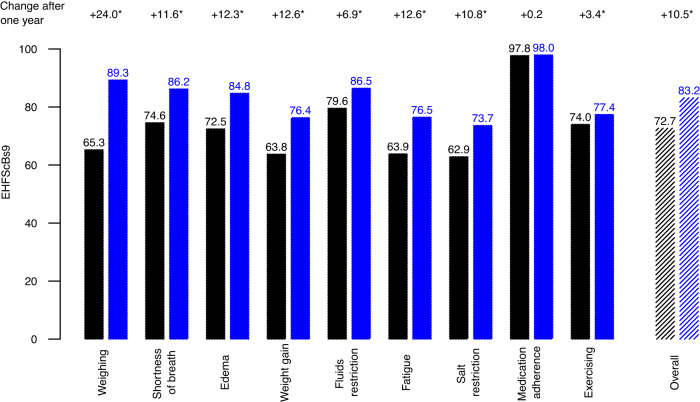
Self-Care Behaviour (EHFScBS-9) at start of programme (black) compared to follow-up after 1 year (blue). Values standardised to 0–100, with 100 meaning full agreement. The numbers at the top indicate the change after one year. The star indicates statistically significant differences between start of programme and 1-year follow-up with a *p*-value of <0.001 (paired Wilcoxon tests). Black: Start of programme. Blue: After one year. Questions: 1: I weigh myself every day, 2: If shortness of breath increases, I contact my doctor or nurse, 3: If my legs/feet are more swollen, I contact my doctor or nurse, 4: If I gain weight more than 2 kg in 7 days, I contact my doctor or nurse, 5: I limit the amount of fluids (no more than 1.5–2 litres a day), 6: If I experience fatigue, I contact my doctor or nurse, 7: I eat a low-salt diet, 8: I take my medication as prescribed, 9: I exercise regularly.

The participants’ self-care was measured using the EHFScBs-9^22^. Paired self-care data at initiation of the telehealth programme and after one year was analysed in a subgroup of 2.358 participants (Fig. 5). The mean standardised aggregated total scores over the nine items increased from 72.8 to 83.2, respectively, resulting in an increase of 10.5 points (*p* < 0.001, paired Wilcoxon-test), well above the clinically relevant change of 5.75^23^.

## Discussion

Participation in a combined telemonitoring and telecoaching programme for ambulatory HF patients recently hospitalised and at high risk for re-hospitalisation was related to a significant decrease in all-cause mortality and a significant reduction in hospitalisation for HF. The programme also led to an significant increase of DAOH. To our knowledge, our study is the largest primary report on a combined telehealth programme in chronic HF patients.

Like other telemedical interventions with positive impact on hospitalisation rates and patients’ mortality^13,14^, the telehealth programme assessed here included thorough telemonitoring, with daily monitoring of key symptoms and signs of HF, immediate data transmission to a telemedical centre as well as daily assessment of those parameters by specialised personnel. This assured rapid detection of relevant changes in vital parameters or patients’ symptoms, prompting specific alerts in case of imminent worsening, rapid contact with the patients and, in some cases, initiation of physician contact. Early identification of a cardiac decompensation allows timely intervention and appeared, in this study, to have translated in an avoidance of subsequent hospitalisations.

On the other hand, we observed an increase in hospitalisations for sleep disorders, ischaemic heart disease, and angina pectoris. This may indicate that patients reported typical symptoms to the telenurses, who recommended – based on standardised procedures – the need for further assessment, possibly resulting in a decrease of subsequent life-threatening events.

Likewise, continuous patient support and education through telecoaching improves patients’ adherence to medical treatment and healthy lifestyle, such as monitoring of salt and fluid intake^24^. Non-adherence to medication regimens is known to increase HF hospitalisations and mortality^25^, and interventions to improve adherence have been proven to have beneficial effect^24,25^. Participants of this study reported high adherence to medication and self-care behaviour to prevent HF decompensations, as reflected by high EHFScB-9 scores at time of inclusion with even an overall increase during the telehealth programme. Thus, improved self-management, including an increased awareness of warning signs and higher adherence to medication, might be another factor driving the effect of the telehealth programme seen in this study.

The effect of a telemedical intervention depends on the patients’ adherence to the programme^26^. A post hoc secondary analysis of the BEAT-HF trial showed that lower adherence to weight monitoring in a given week is associated with an increased risk of subsequent hospitalisation or death in the following week^27^. The adherence of participants to the telemonitoring in our study was high compared to other telemedical intervention studies with negative outcome^16,20^. An easy-to-use telehealth programme such as the one used in this study, with only two monitoring devices might facilitate regular utilisation, whilst complex programmes with multiple devices are more prone to technical errors and might discourage users^24^. This is particularly the case for elderly patients with beginning visual impairment, motoric disabilities or even mild cognitive impairment, common comorbidities in HF patients^24^.

Finally, an explanation for the effect size observed in this study is the focus on a population at high risk for HF re-hospitalisation. In this high-risk cohort the effect of regular telemedical interventions might be greater than in a mixed cohort of ambulatory chronic HF patients with all risk levels, and might explain the differences compared to other studies^5,13^. Similarly, an exploratory subgroup analysis of the neutral TIM-HF trial revealed a reduction of days lost to HF admission in the subgroup of patients with a cardiac decompensation within the 24 months prior^28^. Thus, our study highlights the importance of patient selection for telemedical interventions.

Compared to other randomised controlled trials (TIM-HF, TIMI-HF, IN-TIME), the study population of this trial was considerably older (mean age 75 vs 65 years in IN-TIME and 70 in TIM-HF and TIM-HF2). As age is an important predictor of all-cause mortality among heart failure patients^29^ this might explain the overall higher mortality seen in our study.

The main limitation of our study is its retrospective design. Although propensity score matching allows to balance control and intervention groups with respect to baseline variables, it still relies on retrospective data and selection bias as well as overfitting, and inability to account for unmeasured confounders is possible. The outcome data of this study was obtained from reimbursement data from a German health insurance company, thus being possibly influenced by coding errors and not widely generalisable to other health care systems. However, the results may a good reflection of everyday clinical practice compared to randomised controlled trials, where the control group may not represent *de facto*, but rather optimal clinical care. The study was conducted, in part, during the Covid19 pandemic. A telehealth programme may be of particular value in such times when physical interaction is restricted, which may have contributed to the positive outcome of the study. The major strength of this study is its size; with more than 12,000 patients analysed, this is, to our knowledge, the largest report on telehealth in HF published so far.

In conclusion, studying ambulatory HF patients recently hospitalised and at high risk for re-hospitalisation in a real-life setting, a comprehensive telehealth programme combining telemonitoring and telecoaching led to a significant reduction of all-cause mortality and HF hospitalisations compared to usual care. For an efficient implementation of telemedicine in the clinical future further research is needed to identify 1) patients who particularly benefit from telehealth programmes, 2) drivers of effectiveness of telemedical interventions and influence of adherence, 3) optimal duration and timing of telemedical interventions, 4) adequate complexity of telemonitoring systems, balancing user-friendliness and optimal patient monitoring.

## Methods

### Study design

This study is a retrospective case-control comparison between HF patients participating in a comprehensive telehealth programme (mecor®) from a German statutory health insurer (Knappschaft, Bochum, Germany) and a matched, usual care group derived from patients of the same health insurance provider. In this real-world setting, random assignment of patients to the intervention group was not possible because of legal and logistical constraints. Therefore, we used propensity score matching as described below. All clinical information on mortality, hospitalisation and medication was obtained from reimbursement data. The study was approved by the local ethics committee (Technical Universitiy of Munich, 623/20) and conformed to the ethical standards of the Declaration of Helsinki. It was registered at the German Clinical Trial Register with the identifier DRKS00026197.

### Inclusion and exclusion criteria

The telehealth programme was offered patients who had been hospitalised for HF in the last 18 months and were considered to be at high risk for re-hospitalisation by their health insurer. The health insurer identified possible candidates using claims data on diagnoses, previous hospital stays, and prescriptions estimating the risk of re-hospitalisation in the subsequent year using the adaptable, comprehensive, risk assessment methodology to obtain the likelihood of hospitalisation (ACRA-LoH).

Patients under 40 years of age or patients suffering from severe impaired hearing or sight, dementia, schizophrenia, dependency syndromes, severe chronic kidney disease (stage 4 and 5), terminal HF, or with very high level of nursing care requirement were not eligible to participate (see Supplementary Table 4 for detailed inclusion and exclusion criteria).

Patients remained in the programme until they were unable to continue the programme (e.g., due to death, change of insurance company, or development of one of the exclusion criteria) or voluntarily withdrew (see supplementary results for a detailed description of dropouts).

### Calculation of the risk of hospitalisation

The likelihood of hospitalisation (LoH) was calculated based on historical data of patients insured by the health care insurance with the ACRA methodology. The historical patient data included claims data on previous hospital stays, diagnoses (ICD-codes), procedures (OPS-codes, according to the German Operationen- und Prozedurenschlüssel), and prescriptions (ATC codes, according to the Anatomical Therapeutic Chemical Classification System). Using this historical administrative and claim data, statistically relevant predictive parameters for hospitalisation and their weights were identified by LASSO (least absolute shrinkage and selection operator) regression. A 12-fold cross-validation was performed. The model was first implemented in November 2017 and featured 2.893 from a total of 31.648 possible factors, resulting in an area under the curve (AUC) of 0.6825. The individual factors were categorised into four main subsets: drug prescriptions, previous and current diagnoses, procedures or operations and hospitalisations’ data, including length and frequency of stays. The model was recalculated in September 2019 and May 2020, resulting in an AUC of 0.6948, and of 0.6885, respectively. Candidate selection from all patients insured at the participating health insurance was performed regularly (11/2017, 06/2018, 11/2018, 07/2019, 09/2019, 12/2019, 05/2020, 09/2020) and for each candidate selection, the most recent model was used.

Patients with an ACRA-LoH of 37.75% or higher were considered to be at high risk for recurrent hospitalisation. This threshold was believed to constitute the economic break-even point for the programme.

### Identification of study participants

Suitable candidates for the telehealth programme were identified at multiple time points (11/2017, 06/2018, 11/2018, 07/2019, 09/2019, 12/2019, 05/2020, 09/2020). In total, 119,715 individual patients diagnosed with HF but not fulfilling any exclusion criteria were identified. Of these, 89,239 were considered at high risk for re-hospitalisation within one-year (according to ACRA-LoH) and thus were considered possible candidates for enrolment in the telehealth programme. Due to resource constraints of the telemedical centre, these possible candidates were prioritised for participation in the telehealth programme by the main diagnosis of their last hospitalisation (group 1: HF: *n* = 26,613; group 2: other cardiovascular diseases: *n* = 31,511; group 3: all others: *n* = 31,115) and were successively invited to participate in the telehealth programme by letter, followed by a telephone call to respond to any questions about the programme. Of the 89,239 possible candidates, 13,050 were no longer available for participation because they died prior to enrolment, 636 left the insurance company and 16,217 declined participation. Out of the remaining 59,336 possible candidates, 6065 enroled in the programme, leaving 53,271 possible candidates served as potential controls for matching (Fig. 1).

Enrolment in the telehealth programme was voluntary; all participants gave written informed consent to participate in the telehealth programme, including data collection and presentation for research purposes. Personal data was processed in accordance with Directive 95/46/EC (GDPR; see supplementary notes).

### Telehealth programme

The telehealth programme consisted of daily remote telemonitoring of HF signs/symptoms and individualised telecoaching sessions (mecor® telehealth programme). Telecoaching sessions on disease related topics (e.g., warning signs of decompensation, adherence to medication, fluid restriction, physical activity) to support patients’ self-management were conducted by telenurses specifically trained for HF on a 1:1 basis every 4–12 weeks, according to the participants’ individual needs and knowledge. The workflow was driven by the patient management software with algorithms accounting for distinct risk, clinical profile, coping, and resources of the patient. Telemonitoring involved daily assessment of the patients’ weight and HF symptoms and daily transmission of the data to the telemedical centre. Patients’ symptoms were assessed through the following questions: changes in 1) shortness of breath, 2) cough, 3) tiredness/fatigue, 4) lower extremity oedema, and 5) need for an additional pillow at night. The answers were captured with a telemonitoring device (Tunstall RTX3371). Patients’ weight was monitored with a digital scale (Fairbanks HCS Telescale) and automatically transferred via Bluetooth technology to the telemonitoring device. All data was automatically transmitted to the telemedical centre via mobile network. Age-appropriate and easy-to-use technology (e.g., audio output of system, large screen, large haptic buttons) were used to optimise usability.

In the telemedical centre, patients’ data was stored on a secure server and analysed in a step-wise approach: first, the data was automatically checked for validity, then, several pre-specified rules were automatically applied to identify imminent decompensation (e.g., weight gain, repeated worsening of symptoms, no data entry for 3 consecutive days, exceeding individualised thresholds). In case of a rule violation an alert was raised by the IT systems. A trained telenurse reviewed the alert by the end of the same working day (if the IT alert entered after 4.30 PM, within the end of the next working day). The nurse contacted the patients by telephone using a structured and standardised interview to verify the validity of the alarm and to assess the patients’ condition. The telenurses advice also followed an established protocol with escalating behavioural interventions, but also considered individual patients’ factors, severity of symptoms and velocity of onset, as well as the personal experience. Instructions include general behaviour changes (e.g. reduction of salt or fluid intake, recommendation of exercise or rest), adherence to the prescribed medication regimen, taking standby or on demand (diuretic) medication, and even seeking physician advice (primary care, cardiologist or emergency department, depending on the patient condition).

### Identification of the usual care control group via propensity score matching

Out of the available 59,336 possible candidates, 6065 participated in the program (see above). From the remaining 53,271 possible candidates a group of equal size was identified in a two-step matching procedure to reduce treatment assignment bias and mimic randomisation with the aim to identify the usual care control group closest to the group of patients participating in the telehealth programme.

In step one, for each participant all potential controls that exactly matched in gender, age group (≤70 years, 70–76 years, 77–81 years, ≥82 years), and the last main hospital diagnosis group were identified. In step two, for each participant out of his exact matches the patient with the proximity score closest to the participant was selected and included in the final control group (nearest neighbour matching). The proximity score reflected the propensity of a candidate to participate in the telehealth programme prior to their decision and hence avoided a selection bias when identifying the UC control group. To calculate the proximity scores, a logistic regression model was derived based on all candidates that agreed to participate or declined participation. This saturated model was estimated using the label “agreed”/ “declined” participation as the dependent variable and the variables identified by the separate ACRA-LoH methodology as independent variables (base model)^30^. Sensitivity analyses confirmed the quality of the matching (see supplementary methods). We also studied outcome in those who declined participation in the telehealth programme (see supplementary methods).

### Endpoints

The primary endpoint was all-cause mortality. Secondary endpoints included: 1) number of hospitalisations for HF, for cardiovascular causes and all-cause hospitalisations, 2) days hospitalised and days alive outside hospital (DAOH), 3) adherence to the telemonitoring programme and change in self-care behaviour.

DAOH were calculated as the number of days spent alive and out of hospital divided by the intended follow-up time. Adherence to the telemonitoring programme was defined as the ratio of days on which telemonitoring data from the patients was received to the days on which telemonitoring device data was expected. The participants’ self-care behaviour was measured using the European Heart Failure Self-care Behaviour Scale (EHFScBS-9)^22^. The EHFScBS-9 was completed by the participants at the beginning of the combined telemonitoring and telecoaching programme and repeated annually to monitor progress.

### Statistical analyses

All patients who started the telehealth programme between January 1^st^ 2018 and September 30^th^ 2020 were included in this analysis. Evaluation started after a 28-day-training/run-in period to give the patient sufficient time to get acquainted with the telemonitoring system before starting the evaluation. Control patients were matched to each patient in the telehealth programme at the end of the respective run-in-period and continued in their usual care setting. Evaluation ended at the earliest time point out of either the end of the evaluation time frame (31^st^ December 2020), death or termination of the insurance contract (intention-to-treat-analysis). A secondary on-treatment-analysis evaluated only the period of a patient’s participation in the telehealth programme.

The statistical software R (Version 3.6.2) was used for all analyses. Cumulative survival curves for time-to-event analyses (death) were based on Kaplan–Meier estimates. Hazard ratios were analysed relying on Cox proportional hazards models, with treatment as the only explanatory variable. For time-to-event analyses competing risks were considered. Cumulative incidence function was used to estimate the marginal probability of first hospitalisation due to either HF, cardiovascular cause or all-cause with death as the competing risk. Number of hospitalisations and hospital days were modelled using a negative binomial regression, with treatment and ACRA-LoH as explanatory variables and evaluation length as offset variable.

Confidence intervals (CI) for the DAOH were bootstrapped-based. A two-sided t-test was used to compare DAOH between the groups. For statistical analyses of the EHFScBS-9, the initial survey was compared to the survey after one year in the telehealth programme. In accordance with the survey specifications, in case of at most three missing items, the missing item scores were set to three^22^. All single item scores and the aggregated overall scores were reverse standardized to 0–100 by linear transformation, i.e., a higher score indicating better self-care^23^. Paired Wilcoxon tests were used to compare the single item- and overall scores over time.

We considered a *p*-value < 0.05 to result in statistically significant differences. Statistically significant results remained so, after applying the Benjamini–Hochberg-procedure for multiple comparisons unless noted otherwise.

### Reporting summary

Further information on research design is available in the Nature Research Reporting Summary linked to this article.

### Supplementary information

Supplement

Reporting Summary

## Data Availability

The data that support the findings of this study are available upon reasonable request from the authors (K.K., C.K., S. Sc.). The data are not publicly available due to state restrictions (participant privacy/consent).
