# Supplementary material for: Combined telemonitoring and telecoaching for heart failure improves outcome
Source: NPJ Digit Med. 2023 Oct 17;6:193. doi: 10.1038/s41746-023-00942-4 (PMC10582035; doi:10.1038/s41746-023-00942-4)
Supplement: Supplementary file 1 — Supplement [file 41746_2023_942_MOESM1_ESM.pdf]

Supplementary Information

Supplementary Methods: Sensitivity Analysis

Sensitivity analysis for the identification of the usual care control group

For the matching of the control group the proximity score was calculated as described above. For sensitivity analyses, two additional matching scores were calculated using 1) a score based on a LASSO regression model using all available variables from historic payer data as independent variables, and 2) the ACRA-LoH scores previously obtained (see above). The sensitivity analyses yielded a similar quality of the matching as the matching used in the main analysis (Supplementary table 1 and 2). Therefore, the base matching model was used for the outcome analysis.

Sensitivity analysis for the mortality of patients declining participation

To exclude a possible bias in identification of the usual control group, we analysed the all-cause mortality after one year in patients who were asked to participate in the telehealth programme and declined participation. The mortality probability of decliners (11.3%, CI: 10.8 – 11.9) and patients in the usual care group (11.0% CI: 10.1 – 11.9) was similar, and higher than of patients in the telehealth group (4.8%, CI: 4.2 – 5.4). However, the group of patients who declined participation was older (mean age 80.4 years in decliners, 75.4 in the usual care group and 75.3 in the telehealth group) and had more females (51% males in decliners and 62.7% in both the usual care and telehealth group). Patients´ morbidity, assessed through the likelihood of hospitalisation (ACRA-LoH) was similar in all three groups (53.9% in decliners, 55.5% in the usual care and 54.3% in the telehealth group).

Supplementary Table 1

Metric	Telehealth intervention group	Usual care group	Cohen's d
Number of patients	6,065	6,065	-
Dimensions exactly matched			
Gender (male)	3800 (62.7%)	3800 (62.7%)	-
Age Groups			
younger than 70 years	1,469 (24.2%)	1,469 (24.2%)	-
70 to 76 years	1,458 (24.0%)	1,458 (24.0%)	-
77 to 81 years	1,710 (28.2%)	1,710 (28.2%)	-
82 years and older	1,428 (23.5%)	1,428 (23.5%)	-
Main diagnosis at hospitalisation immediately preceding screening			
Heart failure [a]	2,831 (46.7%)	2,831 (46.7%)	-
Cardiovascular excluding heart failure [b]	2,141 (35.3%)	2,141 (35.3%)	-
All other	1,093 (18.0%)	1,093 (18.0%)	-
Proximity scores			
ACRA-LoP (likelihood of participation)	37.4% ± 14.0 %	37.3 % ± 13.8 %	.010
Baseline characteristics not used for matching			
Age in years	75.3 ± 8.9	75.2 ± 9.0	.009
Hospitalisations, during the last 12 months prior to screening			
Number of all-cause hospitalisations	2.14 ± 1.64	2.29 ± 1.74	.087
Number with main diagnosis heart failure[a]	0.46 ± 0.67	0.45 ± 0.68	.015
Number of all hospital diagnoses (main and secondary diagnoses)	23.8 ± 22.0	26.0 ± 23.3	.100
Time since last preceding hospitalisation	161 ± 110	148 ± 107	.118
Days in hospital during previous 12 months	18.8 ± 20.6	20.3 ± 21.4	.072
Medication during the last 12 months prior to screening			
Number of prescription	87.2 ± 48.1	91.8 ± 49.2	.095
Proportion with ACE-inhibiters or ARBs	84.3%	84.1%	.007
Proportion with ARNI	7.5%	6.8%	.026
Proportion with Betablocker	83.0%	82.2%	.022
Proportion with Diuretics	84.2%	83.9%	.008
Proportion with MRA	33.9%	33.1%	.018
Comorbidities during the last 12 months prior to screening			
Hypertension	82.6%	84.7%	.058
Coronary heart disease	55.9%	57.5%	.032
Stroke	2.1%	2.5%	.023
Kidney disease	39.8%	43.5%	.075
Diabetes mellitus	30.4%	33.7%	.071
COPD and/or Asthma	48.6%	52.5%	.078
Malignant diseases	8.1%	10.1%	.070

Supplementary Table 1: First Sensitivity Analysis. Matching score for UC group based on LASSO regression model over all available variables. Values are presented as absolute numbers ± standard deviation of as proportions (in %). a) Heart failure defined as ICD-10 Codes I50., I11.0*, I13.0*, I42.0*. b) Cardiovascular excluding heart failure defined as ICD-10 Codes I* excluding ICD-10 Codes associated to heart failure. Cohen's d is a measure of effect size where values <0.2 indicate small effects.*

Supplementary Table 2

Metric	Telehealth intervention group	Usual care group	Cohen's d
Number of patients	6,065	6,065	-
Dimensions exactly matched			
Gender (male)	3800 (62.7%)	3800 (62.7%)	-
Age Groups			
younger than 70 years	1,469 (24.2%)	1,469 (24.2%)	-
70 to 76 years	1,458 (24.0%)	1,458 (24.0%)	-
77 to 81 years	1,710 (28.2%)	1,710 (28.2%)	-
82 years and older	1,428 (23.5%)	1,428 (23.5%)	-
Main diagnosis at hospitalisation immediately preceding screening			
Heart failure [a]	2,831 (46.7%)	2,831 (46.7%)	-
Cardiovascular excluding heart failure [b]	2,141 (35.3%)	2,141 (35.3%)	-
All other	1,093 (18.0%)	1,093 (18.0%)	-
Proximity scores			
Prospective one-year LoH (ACRA-LoH)	54.3% ± 14.5 %	54.3 % ± 14.4 %	.000
Baseline characteristics not used for matching			
Age in years	75.3 ± 8.9	75.7 ± 9.2	.044
Hospitalisations, during the last 12 months prior to screening			
Number of all-cause hospitalisations	2.14 ± 1.64	2.05 ± 1.69	.053
Number with main diagnosis heart failure[a]	0.46 ± 0.67	0.42 ± 0.65	.067
Number of all hospital diagnoses (main and secondary diagnoses)	23.8 ± 22.0	23.3 ± 22.4	.022
Time since last preceding hospitalisation	161 ± 110	168 ± 113	.065
Days in hospital during previous 12 months	18.8 ± 20.6	18.9 ± 21.4	.007
Medication during the last 12 months prior to screening			
Number of prescription	87.2 ± 48.1	86.5 ± 47.2	.014
Proportion with ACE-inhibiters or ARBs	84.3%	83.5%	.022
Proportion with ARNI	7.5%	6.1%	.056
Proportion with Betablocker	83.0%	81.4%	.042
Proportion with Diuretics	84.2%	82.8%	.038
Proportion with MRA	33.9%	32.3%	.035
Comorbidities during the last 12 months prior to screening			
Hypertension	82.6%	80.2%	.062
Coronary heart disease	55.9%	51.7%	.086
Stroke	2.1%	2.1%	.003
Kidney disease	39.8%	39.5%	.007
Diabetes mellitus	30.4%	30.1%	.006
COPD and/or Asthma	48.6%	46.8%	.036
Malignant diseases	8.1%	10.5%	.083

Supplementary Table 2: Second Sensitivity Analysis. Matching score based on ACRA-LoH score. Values are presented as absolute numbers ± standard deviation of as proportions (in %). a) Heart failure defined as ICD-10 Codes I50., I11.0*, I13.0*, I42.0*. b) Cardiovascular excluding heart failure defined as ICD-10 Codes I* excluding ICD-10 Codes associated to heart failure. Cohen's d is a measure of effect size where values <0.2 indicate small effects.*

Supplementary Table 3

Socioeconomic characteristics of the telehealth intervention group		
	n= 6,065	%
Occupation/ Profession	n	
Stay-at-home parent/ partner	198	3.3
Unskilled labourer/ auxiliary worker	207	3.4
Craftsperson / labourer/ clerk	2.727	45.0
Skilled worker/ mid-level employee	2.216	36.5
University graduate/ executive employee	717	11.8
Education		
No school graduation/degree	281	4.6
General secondary school degree (Hauptschule)	3.721	61.4
High school degree (Realschule)	1.193	19.7
College degree (Gymnasium/Abitur)	428	7.1
University degree	442	7.3
Marital status		
Single	168	2.8
Life partnership	247	4.1
Married	3.675	60.6
Divorced	350	5.8
Widowed	1.625	26.8
Social contacts (family and friends)		
Good (at least weekly)	4.258	70.2
Medium (at least monthly)	1.041	17.2
Bad (less than once per month)	766	12.6
Financial status		
No financial burden	3.372	55.6
Small financial burden	1.113	18.4
Average financial burden	1.105	18.2
Significant financial burden	475	7.8

Supplementary table 3: Socioeconomic characteristics of the telehealth intervention group

Supplementary Results: Description of study dropouts

During the evaluation period, 1,101 patients from the intervention group left the programme (dropouts). Of those, 665 patients decided to leave the programme without a medical reason (e.g., due to programme fatigue, resignation with their disease; average participation 207 days, IQR 73 to 294 days); 191 patients developed exclusion criteria while on the programme (e.g., moved to nursing home, new onset of chronic kidney disease stage 4 or 5; average participation 325 days, IQR 130 to 488 days); 138 withdrew because of health deterioration (e.g., onset of new diseases like cancer; average participation 245 days, IQR 113 to 348 days); 56 patients were non-compliant (average participation 327 days, IQR 191 to 427 days; non-compliance for the purpose of programme was defined as a patient missing a scheduled telecoaching session and not responding to at least 3 subsequent telephone calls and 2 letters over 6 weeks with the attempt to reschedule the call); 5 patients had technical issues with the equipment (average participation 178 days, IQR 89 to 189 days) and 46 patients did not give any specific reason for withdrawing (average participation 243 days, IQR 87 to 347 days). Overall, the average time of participation in the telehealth programme was 240 days (IQR 88 to 349 days) for the patients that dropped-out and 486 days (IQR 297 to 637 days) for patients not withdrawing from the programme before evaluation. For the intention-to-treat analysis (ITT), all patients (including the dropouts) were evaluated as long as they could be followed-up, i.e., as long as they remained insured at the participating insurance company, while for the on-treatment (OT) analysis the evaluation ended with the dropout.

Supplementary Figure 1: On-treatment analysis

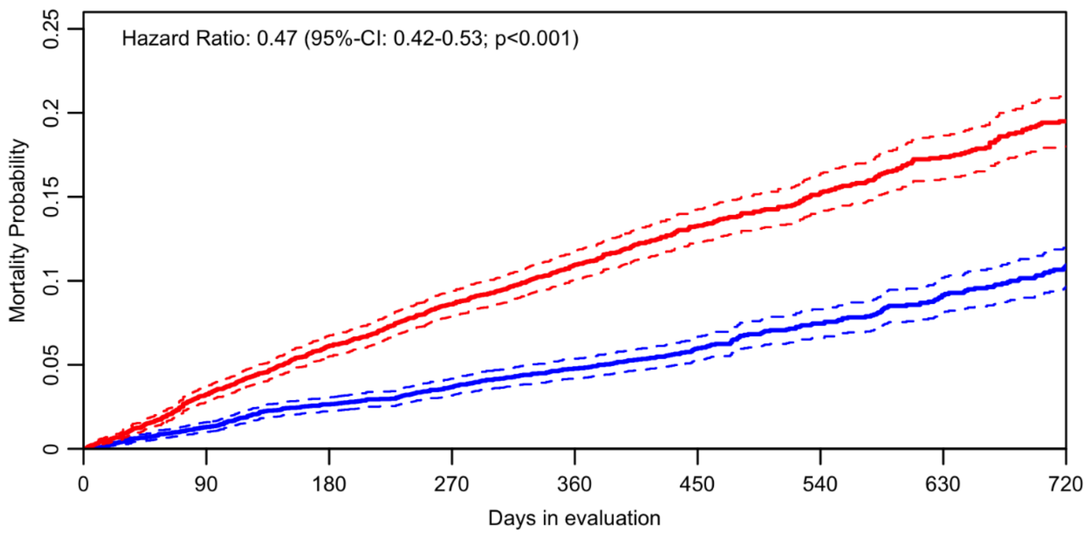

Patients at risk (events in 90-day period)	0	90	180	270	360	450	540	630	720
TH group	6065	5706 (77)	5189 (75)	4394 (51)	3589 (46)	2359 (38)	1790 (32)	1368 (29)	1041 (23)
UC group	6065	5591 (192)	4999 (160)	4156 (122)	3345 (97)	2172 (75)	1616 (42)	1220 (36)	928 (28)

TH Group UC Group

Supplementary figure 1: Kaplan-Meier-plots of the all-cause mortality probability (continuous line) with 95%-confidence interval (dotted line) in the on-treatment-analysis. Hazard ratio 0.47, 95%-confidence interval: 0.42-0.53, p<0.001, Wald-test. Blue line: Telehealth group. Red line: Usual care group.

Supplementary Table 4: Detailed inclusion and exclusion criteria

Inclusion criteria	Exclusion criteria
• Hospital discharge with heart failure diagnosis (ICD-10-GM I50*, I11.0*, I13.0*, I42.0) within the last 18 months<br>• Likelihood of hospitalisation ≥37.75% within the next 12 months<br>• ≥40 years of age	• Chronic kidney disease stage 4 or 5 (N18.4 or N18.5)<br>• Cardiac assist device (Z95.80)<br>• Dementia (F00*-F03*)<br>• Dependency syndromes (F10.2, F11.2, F12.2, F13.2, F14.2, F15.2, F16.2, F19.2)<br>• Schizophrenia (F20-F29)<br>• Alzheimer disease (G30.0, G30.1, G30.8, G30.9)<br>• Lack of legal capacity<br>• Nursing home residence or very high level of nursing care requirement<br>• Severely impaired hearing or sight

Supplementary table 4: Detailed inclusion and exclusion criteria

Supplementary Notes: Data protection and information security

For this study, personal data from patients participating in the mecor® tele-health programme (participant data) were analysed. For these data, Health Care Systems GmbH (HCSG) is the data controller within the meaning of Article 4 (7) of Regulation (EU) 2016/679 of the European Parliament and of the Council of 27 April 2016 (General Data Protection Regulation, GDPR). HCSG remains solely responsible for the processing of participant data in accordance with Article 24 GDPR.

Before participating in the telehealth programme, patients gave their explicit consent to the processing of their personal data according to Articles 6 (1) (a) and 9 (2) (a) GDPR. At the time of enrolment into the telehealth programme, patients were informed according to Article 13 GDPR. The information comprised, for example, details about the data controller, the participants' rights under GDPR, the purpose of data processing together with categories, types and sources of data. In particular, patients were informed that their data may also be processed for the purpose of research.

Patients' electronic health records are kept on the mecor software. The information security management system of HCSG is certified according to ISO/IEC 27001.

Personal data were extracted and analysed by the data controller' staff in the data controller's highly secure data centre. Investigators that were not part of the data controller's organization were provided with anonymized data for further statistical analyses.
